# *Ulosarcina terrestrica* gen. nov., sp. nov., a New Ulvophycean Sarcinoid Alga from the Russian Far East

**DOI:** 10.3390/plants11233228

**Published:** 2022-11-25

**Authors:** Andrey A. Gontcharov, Arthur Yu. Nikulin, Vyacheslav Yu. Nikulin, Rezeda Z. Allaguvatova, Veronika B. Bagmet, Shamil R. Abdullin

**Affiliations:** Federal Scientific Center of the East Asia Terrestrial Biodiversity, Far Eastern Branch of the Russian Academy of Sciences, 159, 100-Letia Vladivostoka Prospect, 690022 Vladivostok, Russia

**Keywords:** sarcinoid green alga, Ulvophyceae, new genus and species, SSU rDNA, secondary structure, morphological characteristics, life cycle, temperate monsoon climate zone

## Abstract

Many filamentous and sarcinoid terrestrial or freshwater green algae that were previously assigned to the Chlorophyceae are members of lineages belonging to the class Ulvophyceae. One of these lineages is the *Planophila*-clade (Ulotrichales). Some genera in this clade share similar morphology: cell packages forming branched pseudofilaments, uniseriate or sometimes biseriate filaments, often embedded in common mucilage. During a study on soil algal diversity in the temperate monsoon climate zone in Russia (Primorsky Territory, Vladivostok), we isolated a strain of sarcinoid green alga and examined it using an integrative approach. SSU and ITS rDNA sequence data, morphological characteristics, and life cycle features differentiated this strain from closely related genera of the order Ulotrichales and led us to describe it as *Ulosarcina terrestrica* gen. et sp. nov.

## 1. Introduction

One of the most successful groups of algae is the phylum Chlorophyta which inhabits the most contrasting ecosystems on Earth [1]. Three classes of green algae, the Chlorophyceae, Ulvophyceae, and Trebouxiophyceae (UTC), make up the core of the phylum, forming the so-called UTC clade [2]. The class Ulvophyceae accounts for above 2500 species [3] that live mainly in marine habitats, with a smaller number of representatives found in freshwaters and non-aquatic habitats. Recent phylogenetic analyses revealed that a number of filamentous and sarcinoid terrestrial or freshwater green algae classified within the Chlorophyceae comprise distinct lineages in the class Ulvophyceae [4]. One of these is the *Planophila*-clade in the Ulotrichales. Some genera in this clade, namely *Sarcinofilum* Darienko & Pröschold, *Planophila* Gerneck, *Rhexinema* Geitler, *Hazenia* H.C.Bold, *Tupiella* Darienko & Pröschold, and *Vischerioclonium* Darienko & Pröschold, share similar morphology: cell packages with different tendencies to form branched pseudofilaments, uniseriate or sometimes biseriate filaments, often embedded in a common mucilage envelope.

During a study on soil algal diversity in the temperate monsoon climate zone in the Russian Far East (Primorsky Territory, Vladivostok), a strain of sarcinoid green alga was isolated and examined using an integrative approach, including light and confocal laser scanning microscopy, and molecular data. It led us to differentiate this strain from other members of the order Ulotrichales and describe it as a new genus and new species, *Ulosarcina terrestrica*.

## 2. Results

### 2.1. Taxonomic Treatment

*Ulosarcina* A.A. Gontcharov, Sh.R. Abdullin, A.Yu. Nikulin, V.Yu. Nikulin, R.Z. Allaguvatova and V.B. Bagmet gen. nov.

Diagnosis: Vegetative cells solitary or in sarcinoid-like two- or three-dimensional packages, embedded in common mucilage; cells spherical and hemispherical, often with vacuoles. Chloroplast parietal, with a distinct pyrenoid, covered with several starch grains. Nucleus single, not visible using light microscopy.

Reproduction by vegetative division or by biflagellated zoospores without a cell wall. Each cell forms a single zoospore. Sexual reproduction was not observed.

Type species (designated herein): *Ulosarcina terrestrica* A.A. Gontcharov, Sh.R. Abdullin, A.Yu. Nikulin, V.Yu. Nikulin, R.Z. Allaguvatova and V.B. Bagmet, sp. nov.

Differs from related genera (*Rhexinema*, *Sarcinofilum*, *Planophila*, *Hazenia* (*Chamaetrichon*), *Tupiella*, *Vischerioclonium*) by SSU-ITS sequences and the following phenotypic characters: formation of sarcinoid-like three-dimensional packets by vegetative cell division rather than the development of filaments; biflagellated zoospores.

**Figure 1:**
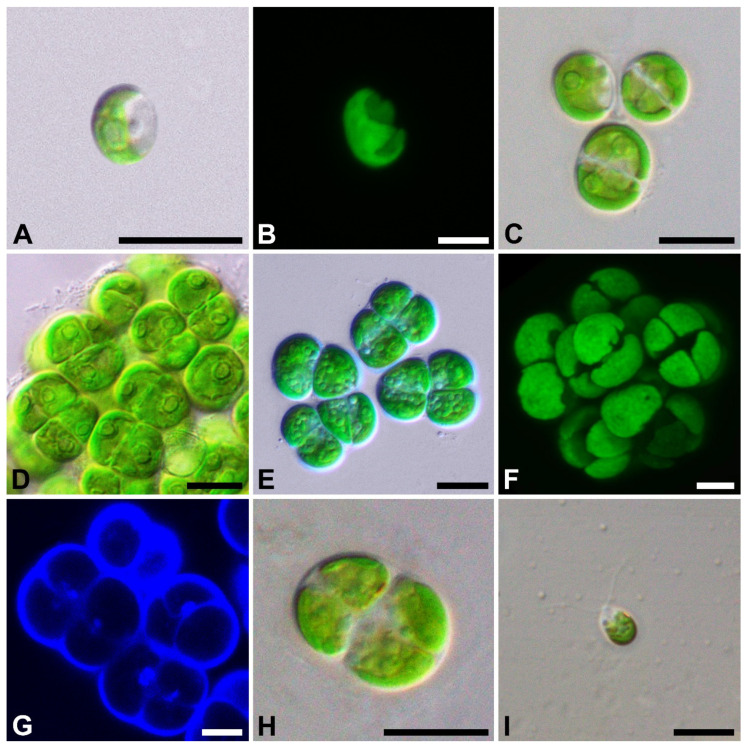
Light micrographs of general morphology (**A**,**C**–**E**,**H**,**I**), confocal reconstruction of chloroplast morphology (**B**,**F**), and confocal optical section of the nucleus (**G**) with bright-field image merged fluorescens channel in *U. terrestrica*. (**A**,**B**) young cells; (**C**) mature solitary cells; (**D**–**F**) mature cells in sarcinoid-like two- and three-dimensional packages; (**G**) cells with a nucleus stained with DAPI; (**H**) zoospores’ formation; (**I**) zoospore. Scale bars: (**A**,**C**–**E**,**H**,**I**) = 10 µm; (**B**,**F**,**G**) = 5 µm.

*Ulosarcina terrestrica* A.A. Gontcharov, Sh.R. Abdullin, A.Yu. Nikulin, V.Yu. Nikulin, R.Z. Allaguvatova and V.B. Bagmet, sp. nov. Figure 1A–I.

Diagnosis: Young cells solitary, spherical, drop-shaped, ovoid, 6.2–9.0 μm long, 5.7–8.8 μm wide, sometimes with vacuoles. The cell wall is thin. Chloroplast parietal (Figure 1A,B). Mature vegetative cells solitary or in sarcinoid-like two- or three-dimensional packages, embedded in common thin homogeneous mucilage (Figure 1C–F; Figure S1), spherical and hemispherical, 8.9–13.3 μm long, 7.6–12.8 μm wide, often with vacuoles. Chloroplast parietal (Figure 1C–F) with a distinct pyrenoid covered with several starch grains (Figure 1C,D). The nucleus is elliptical or subspherical, often lateral, not visible with light microscopy, 1.7–2.2 μm long, 1.4–2.1 μm wide (Figure 1G).

Reproduction by vegetative division (Figure 1C–E) or by spherical to ovoid biflagellated zoospores without a cell wall, 5.5–11.0 μm long, 4.0–8.8 μm wide. Zoospores, with two equal anterior flagella, a parietal chloroplast, a lateral stigma, and vacuoles. The flagella are about as long as the cell (Figure 1I). Each cell produces a single zoospore that is released by gelatinization of the cell wall (Figure 1H). Before stopping, the zoospore moves around its axis and finally loses the flagella. Sexual reproduction was not observed.

Habitat: soil.

Type locality: Russia, Primorsky Territory, Vladivostok (43°11′19.6″ N, 131°55′01.1″ E), in disturbed soil with ruderal vegetation.

Etymology: The species epithet “*terrestrica*“ is based on the habitat where it was found.

Holotype (designated herein): Exsiccatum number VLA-CA-0951, dried biomass of unialgal population was deposited in the Herbarium, Federal Scientific Center of East Asian Terrestrial Biodiversity, Vladivostok, Russia. Gene sequence: DNA sequence obtained from a clonal strain of *U. terrestrica* was deposited in GenBank under accession no. OM700179.

### 2.2. Phylogenetic Analyses

**Figure 2:**
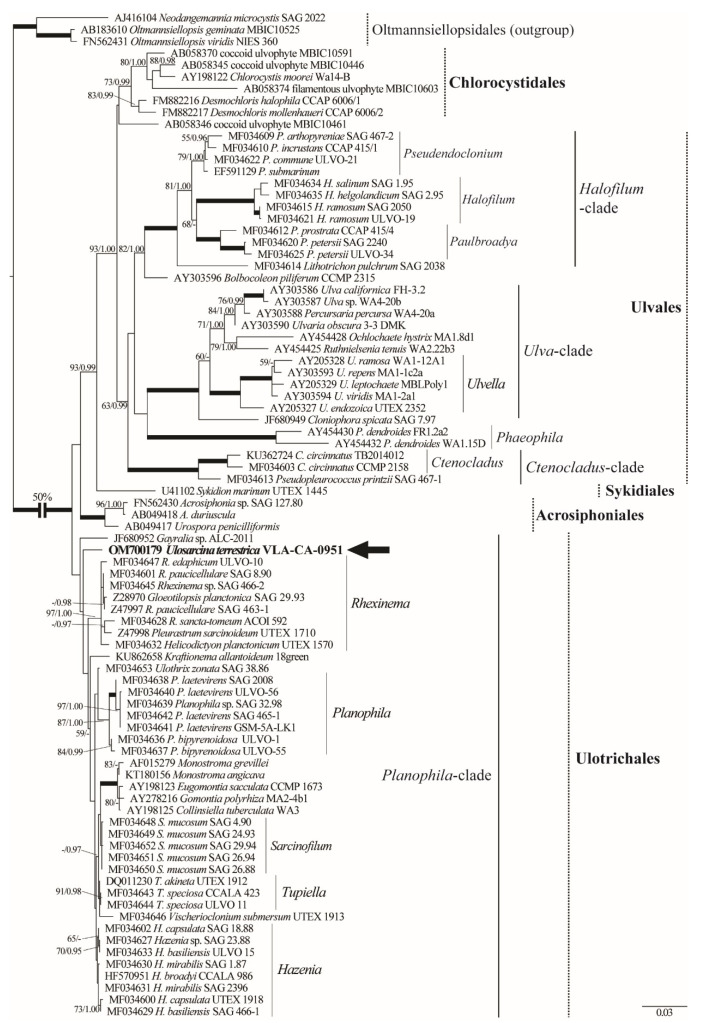
ML phylogenetic tree (TIM2+I+G model) of the Ulvophyceae showing position of the new genus *Ulosarcina* (showed boldface with arrow) based on SSU rDNA sequence data (1771 aligned positions of 86 sequences). The strain designations and GenBank accession numbers of all sequences used in the analyses are given. Support [(BP) ≥ 50% and (PP) ≥ 0.95: ML/BI] are provided above/below the branches. Branches with 100% BP and 1.00 PP are shown in boldface. Clade designations follow Darienko and Pröschold [4] and Darienko et al. [5].

Phylogenetic analyses of 86 SSU rDNA sequences representing major groups of the Ulvophyceae [4] placed the new strain as a member of the Ulotrichales (Figure 2). *Ulosarcina* was poorly resolved in the *Planophila*-clade. Topologically, it branched basally following *Gayralia* sp. (ALC-2011), which was the first divergence in the clade and was the outgroup to the remaining *Planophyla*-clade. Overall resolution in the order Ulotrichales was weak in contrast to the well-resolved relationships in Ulvales clade that was characterized by significantly more divergent SSU rDNA sequences.

**Figure 3:**
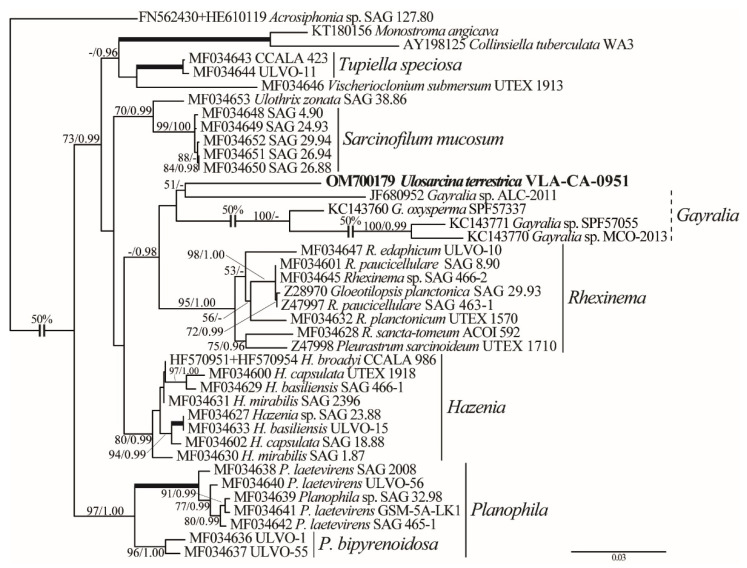
ML phylogenetic tree (TIM2+I+G model) of the Ulotrichales showing the position of the new genus *Ulosarcina* (boldfaced) based on SSU and ITS rDNA sequence data (2291 aligned positions of 40 sequences). See Figure 2 legend for details.

Phylogenetic analyses of the concatenated SSU and ITS rDNA dataset clarified the position of *Ulosarcina* in the Ulotrichales (Figure 3). Divergent ITS sequences added phylogenetic signal, and the tree was much better resolved. *Ulosarcina* showed a weak affinity to *Gayralia* (represented by one SSU rDNA sequence and three not overlapping with it ITS sequences), and this lineage was resolved as a sister (0.98 PP) to a strongly supported *Rhexinema* clade (95/1.00). ITS data along produced almost identical topology and supports (Figure S2). *Sarcinofilum mucosum*, *Monostroma/Collinsiella*, *Tupiella speciosa*, *Hazenia*, and *Planophila* formed moderately to well-supported clades of the tree.

Intrageneric and intergeneric *p*-distances (SSU rDNA) between ulotrichalean genera ranged from 0 to 0.56 ± 0.10% and from 0.23 ± 0.12 to 3.49 ± 0.43%, respectively (Table S1). The divergence was sufficiently higher for the ITS1–5.8S–ITS2 region: 0.48 ± 0.21–6.68 ± 0.71% (intrageneric) and 6.85 ± 1.04–26.18 ± 1.98% (intergeneric *p*-distances). ITS sequence of the new strain differed from those in other genera by more than 16%, which exceeded the difference between some genera: e.g., *Monostroma* and *Collinsiella*—14.75 ± 1.58%; *Vischerioclonium* and *Tupiella*—13.96 ± 1.32%; *Hazenia* and *Ulothrix*—10.91 ± 1.23%; *Sarcinofilum* and *Ulothrix*—6.85 ± 1.04%, etc. The results of the sequence comparisons support our conclusion that *Ulosarcina* is a new genus.

The relatively long branch of *U. terrestrica* in the SSU rDNA-based tree reflected the presence of a significant number of autapomorphic substitutions in its sequence. To locate these substitutions in the SSU rDNA secondary structure and assess their effect on this structure, we compared SSU models of *U. terrestrica* and *Ulothrix zonata* (SAG 38.86; Figure S3). SSU rDNA in *U. terrestrica* had 3 introns of 417 bp, 423 bp, and 449 bp long, located after helix 33, before helix 38, and in helix 50, respectively, and 25 base changes. Of these, 10 substitutions formed 3 CBCs and 4 hCBCs that maintained the secondary structure and 10 substitutions were located in single-stranded (loop) regions and did not alter common secondary structure. Overall, SSU rDNA structure of the new species was the same as in other Ulotrichales.

## 3. Discussion

Phenotypic features observed in the new algal strain isolated from soil in the temperate monsoon climate zone of the Russian Far East (Vladivostok, Russia) such as sarcinoid habit, cell shape, parietal chloroplasts with a pyrenoid, and asexual reproduction by vegetative division and zoospores occur in many groups of green algae and do not allow unambiguous taxonomic assignment even at the class level. Packet-like colonies are known for most classes of green algae, although the number of genera characterized by this morphology is rather limited. Multiple independent origins of this habit in green algae are very likely. Phylogenetic analyses assigned our strain to the order Ulotrichales of the class Ulvophyceae. This assignment further extended the number of non-aquatic algae sharing similar morphology (cell packages with a tendency to form branched pseudofilaments or filaments, often embedded in common mucilage) in the class that mostly accommodated numerous marine taxa until recently [4].

Representatives of Ulotrichales are highly diverse in their cytology, morphology, and ecology, ranging from single-celled organisms to larger multicellular seaweeds [6]. Vegetative cells can divide to form sarcinoid-like two- or three-dimensional structures (a feature of the *U. terrestrica*) in its genera *Planophila*, *Rhexinema*, and *Sarcinofilum*. *Ulosarcina terrestrica* showed no affinity to generic clades sharing similar morphology to any other lineages; or occupied and unresolved position in the order according to rDNA sequence comparison results. In analyses based on a combined SSU and ITS rDNA sequence data, *U. terrestrica* was allied with paraphyletic *Gayralia* (Figure 3). This relationship should be treated with caution because *Gayralia* accessions were represented in the dataset either by SSU or by ITS rDNA sequences only which may have influenced the tree topology. Moreover, these two genera differ profoundly in thallus habit. *Gayralia* has macroscopic parenchymatous gametophytes, initially saccate, later forming leafy monostromatic blades attached to the substrate by rhizoidal protuberances, or they are free floating. The blade-shaped thallus is one cell thick except in the region of the holdfast. *Gayralia* is widely distributed in saline to brackish waters with no reports from non-aquatic habitats (Table S2, based on data [4,7]).

The moderately supported affinity between *Ulosarcina* and *Rhexinema* in the analyses without *Gayralia* (results not shown) is more plausible because these genera are more similar morphologically and occur in non-marine habitats. *Rhexinema* is characterized by very short (2 to 10 cells) filaments or two-dimensional cell packages embedded in common mucilage and reproduction by vegetative division or biflagellated zoospores with stigma. Similar two- and three-dimensional cell packages in common mucilage are also typical for *U. terrestrica,* but no filament formation was observed in this alga. Biflagellated zoospores differentiate *Ulosarcina*, *Rhexinema*, and *Gayralia* (see discussion above) from the rest of Ulotrichales because either quadriflagelated zoospores are known in other genera, or no data on zoospore morphology is available. However, in all phylogenetic analyses, *Ulosarcina* was significantly positioned outside the well-supported *Rhexinema* clade.

## 4. Materials and Methods

### 4.1. Strain Origin, Culture Conditions, and Light Microscopy

A soil sample was collected from the wasteland with ruderal vegetation in Vladivostok city (Primorsky Territory, Russia; 43°11′19.6″ N, 131°55′01.1″ E) on 05 August 2018. Sampling was carried out using standard methods [8]. A strain of sarcinoid green algae was isolated from this sample using the micro-pipette method [9] and cultured in liquid nutrient medium Waris-H [10] at 20–22 °C with a photon fluence 17.9–21.4 μmol photons·m^−2^s^−1^ in a 16:8 h light: dark cycle. The strain was maintained in the culture collection of the Laboratory of Botany in the Federal Scientific Center of East Asian Terrestrial Biodiversity, Russian Federation (strain number VCA-205).

The morphology of vegetative and reproductive cells was examined using an Olympus BX 53 light microscope (Olympus Corporation, Tokyo, Japan) equipped with Nomarski DIC optics and Olympus DP27 digital camera (Olympus Corporation, Tokyo, Japan). Cultures were repeatedly examined throughout lifecycle stages, i.e., in cultures of different ages after transfer.

For confocal laser scanning microscopy, 0.01% Triton X-100 was added to the culture of living algal cells to increase membrane permeability. Then cells were fixed in FAA (3.7%: formaldehyde: 50% ethanol: 5% acetic acid) for 20 min, then rinsed twice and counterstained with DAPI (4,6-diamidino-2-phenylindole, Molecular Probes Inc., Eugene, OR, USA) at a final concentration of 5 µg/mL. After another rinse of samples, fluorescence was detected with LSM 710 LIVE confocal laser scanning microscope (Carl Zeiss, Oberkochen, Germany) at the Instrumental Centre of Biotechnology and Gene Engineering of FSCEATB FEB RAS. DAPI fluorescence was detected at 410–497 nm, and autofluorescence of chloroplasts was recorded in the additional emission channel after 600 nm using Plan-Apochromat 63x/1.40 Oil DIC M27 objective with digital zoom. 3D files of the captured images were recorded and analyzed with ZEN microscope software.

### 4.2. DNA Extraction, Amplification, and Sequencing

For DNA analysis, cultures were harvested during the exponential growth phase and concentrated by centrifugation. Total genomic DNA was extracted as described previously by Abdullin et al. [11]. SSU and ITS rDNA were amplified in two PCR reactions using the primer combinations 82F/N1400R and N920F/ITS055R, respectively [12,13]. PCR was performed using an Encyclo Plus PCR kit (Evrogen, Moscow, Russia) with a T100 Thermal Cycler (Bio-Rad Laboratories, Inc., Hercules, CA, USA) and parameters described by Mikhailyuk et al. [14]. Products were purified using ExoSAP-IT PCR Product Cleanup Reagent (Affymetrix Inc., Santa Clara, CA, USA) and sequenced in both directions using an ABI 3500 genetic analyzer (Applied Biosystems, Waltham, MA, USA) with a BigDye terminator v.3.1 sequencing kit (Applied Biosystems, Waltham, MA, USA) and the same primers used for PCR with additional E528F [15], and ITS03F-800 [16] primers. SSU and ITS rDNA PCR products overlapped for ca. 400 bp, which ensured a non-chimeric concatenated sequence. Sequencing reactions were assembled with the Staden Package v.1.4 [17]. Contig sequence covering partial SSU rDNA and complete ITS region was deposited in GenBank under accession number OM700179.

### 4.3. Alignment, Secondary Structure Modeling, and Datasets

SSU rDNA sequences were aligned according to Darienko and Pröschold [4] in the SeaView program [18] using the secondary structure model of *Ulothrix zonata* (SAG 38.86) as a template. Introns, if present, were excluded from the alignment. Alignment of the divergent spacer sequences (ITS1, ITS2) was guided by primary and secondary structure conservation [19] and folding patterns of *Monostroma* sp. (Ush) and *Rhexinema paucicellularis* (SAG 463-1) proposed by Bast [20] and Darienko and Pröschold [4], respectively. The Mfold web server (http://www.unafold.org/mfold/applications/rna-folding-form.php; accessed on 10 November 2022; [21]) was used with the default settings to generate the ITS1 and ITS2 rRNA secondary structures for *U. terrestrica* (Figure S4), that were then visualized using the program VARNA [22].

In order to clarify the phylogenetic position of the new genus, three datasets were used: (i) the SSU rDNA alignment, including 86 taxa and 1771 bp of representatives of the Ulvophyceae and *Oltmannsiellopsis*-clade used as an outgroup; (ii) concatenated dataset of 40 SSU and ITS rDNA sequences (2291 bp); and (iii) ITS rDNA dataset of 39 sequences (539 bp) of the Ulotrichales and its sister lineage Acrosiphoniales [5] used as an outgroup.

### 4.4. Phylogenetic Analysis

Maximum likelihood (ML) analysis was carried out using PAUP 4.0b10 [23]. Bayesian inference (BI) was performed using MrBayes 3.1.2 [24]. In order to determine the most appropriate DNA substitution model for the datasets, the Akaike information criterion (AIC; [25]) was applied with jModelTest 2.1.1 [26]. ML analysis was done using heuristic searches with a branch-swapping algorithm (tree bisection and reconnection). In BI, four runs of four Markov chains were carried out for 4 million generations, sampling every 1000 generations for a total of 4000 samples. Convergence of the two chains was assessed, and stationarity was determined according to the ‘sump’ plot, with the first 1000 samples (25%) discarded as burn-in. The convergence of the stationary distribution was accessed by ESS values (>200) using Tracer v.1.7.1 [27]. The robustness of the ML trees was estimated by bootstrap percentages (BP; [28]) and posterior probabilities (PP) in BI. BP < 50% and PP < 0.95 were not considered. ML-based bootstrap analysis was inferred using the web service RAxML v.7.7.1 (http://embnet.vital-it.ch/raxml-bb/; accessed on 15 September 2022; [29]). MEGA v.7.0.26 [30] was used to estimate interspecific/intergeneric pairwise distances (*p*-distances).

## Data Availability

The data presented in this study are available on request from the corresponding author. In addition, the data that support the findings of this study are openly available in GenBank.

## Supplementary Materials

The following are available online at https://www.mdpi.com/article/10.3390/plants11233228/s1; Figure S1: Light micrograph of cells embedded in common mucilage stained with India ink. Scale bar = 100 µm. Figure S2: ML phylogenetic tree (TIM2+I+G model) of the Ulotrichales showing the position of the new genus *Ulosarcina* (boldfaced) based on ITS rDNA sequence data (39 sequences and 539 aligned positions). See Figure 2 legend for details. Taxa designation follows Darienko and Pröschold [1]. Figure S3: SSU rDNA secondary structure model of *Ulothrix zonata* (strain SAG 38.86, MF034653) and *Ulosarcina terrestrica* (VLA-CA-0951, OM700179). Base changes in the sequence of the new strain are marked by callouts. Figure S4: ITS1 and ITS2 rRNA secondary structure models for *U. terrestrica* based on Mfold predictions. Table S1: Genetic distances (*p*-distances, %) within analyzed genera based on the aligned SSU (1771 positions) and ITS (ITS1–5.8S–ITS2) region (617 positions) rDNA. Standard error estimates are shown above the diagonal. Table S2: Comparison of phenotypic traits characterizing *Ulosarcina* and related genera in the *Planophila*-clade (based on data [4,7], this study).
