# Supplementary material for: Ulosarcina terrestrica gen. nov., sp. nov., a New Ulvophycean Sarcinoid Alga from the Russian Far East
Source: Plants (Basel). 2022 Nov 25;11(23):3228. doi: 10.3390/plants11233228 (PMC9737678; doi:10.3390/plants11233228)
Supplement: Supplementary file 1 [file plants-11-03228-s001.zip › Table_S2.pdf]

Table S2: Comparison of features characterized *Ulosarcina* and related genera in the *Planophila*-clade

Genus	Thallus morphology	Reproduction	Habitat	References
Ulosarcina	Cells **solitary** or in sarcinoid-like three-dimensional **packages** in common mucilage.	**Vegetative cell division** and by **biflagellated zoospores**. Each cell forms a single zoospore.	Soil	This study
Tupiella Darienko & Pröschold	Prostrate and short erected **filaments**. Branching is abundant.	Asexual reproduction by **quadriflagellated zoospores** and **akinetes**.	Non-marine	Darienko & Pröschold, 2017
Vischerioclonium Darienko & Pröschold 2017	Long branched prostrate and erected **filaments.**	By **filaments disintegration**, **quadriflagellated zoospores** lacking a cell wall, and **akinetes**.	Non-marine	Darienko & Pröschold, 2017
Sarcinofilum Darienko & Pröschold 2017	Short uniserial or long pluriserial **filaments**	By **filaments fragmentation** and **quadriflagellated zoospores**. Each cell forms a single zoospore.	Non-marine	Darienko & Pröschold, 2017
Planophila Gerneck 1907	Cells **solitary** or tightly grouped in small (usually 2–8 cells) **colonies** without mucilage envelope.	**Vegetative division**, by **quadriflagellate zoospores** with or without stigma (4–8 per zoosporangia) and **autospores**.	Non-marine (freshwater, soil)	Škaloud et al., 2018
Rhexinema Geitler 1942	Short (2–10 cells) **filaments** or 2-dimensional **cell packages** in mucilage. Branching is rudimentary.	**Vegetative cell division** and **biflagellated zoospores**.	Non-marine (plankton, mud, soil)	Darienko & Pröschold, 2017
Hazenia H.C.Bold 1958	Short branched **filaments** surrounded by mucilage.	**Filaments fragmentation**. Sexual reproduction by **biflagellated isogametes**.	Non-marine	Škaloud et al., 2018
Gayralia K.L.Vinogradova	Gametophyte **macroscopic** and parenchymatous.	Asexual reproduction by **biflagellate zoospores**	In saline to brackish waters.	Škaloud et al., 2018
