# Supplementary figures and images for: Ulosarcina terrestrica gen. nov., sp. nov., a New Ulvophycean Sarcinoid Alga from the Russian Far East
Source: Plants (Basel). 2022 Nov 25;11(23):3228. doi: 10.3390/plants11233228 (PMC9737678; doi:10.3390/plants11233228)

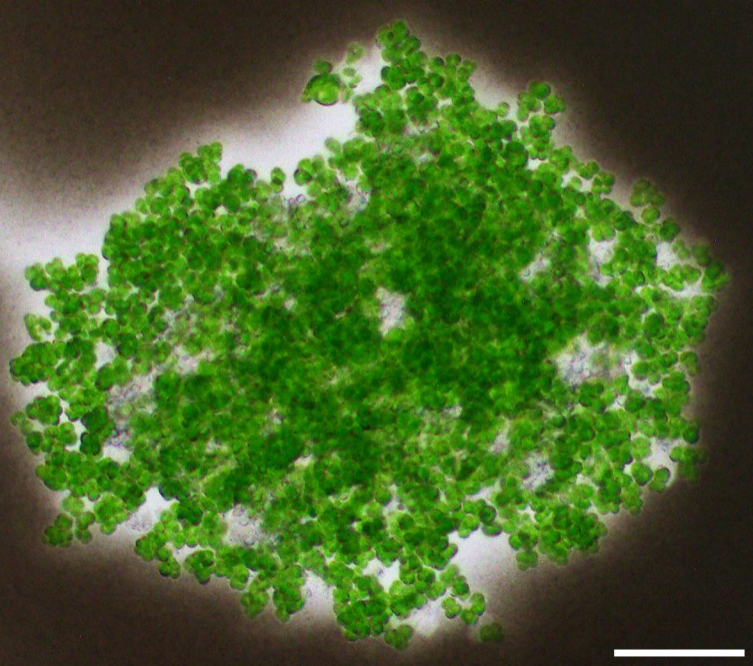

Supplement: Supplementary file 1 [file plants-11-03228-s001.zip › Fig_S1.pdf]

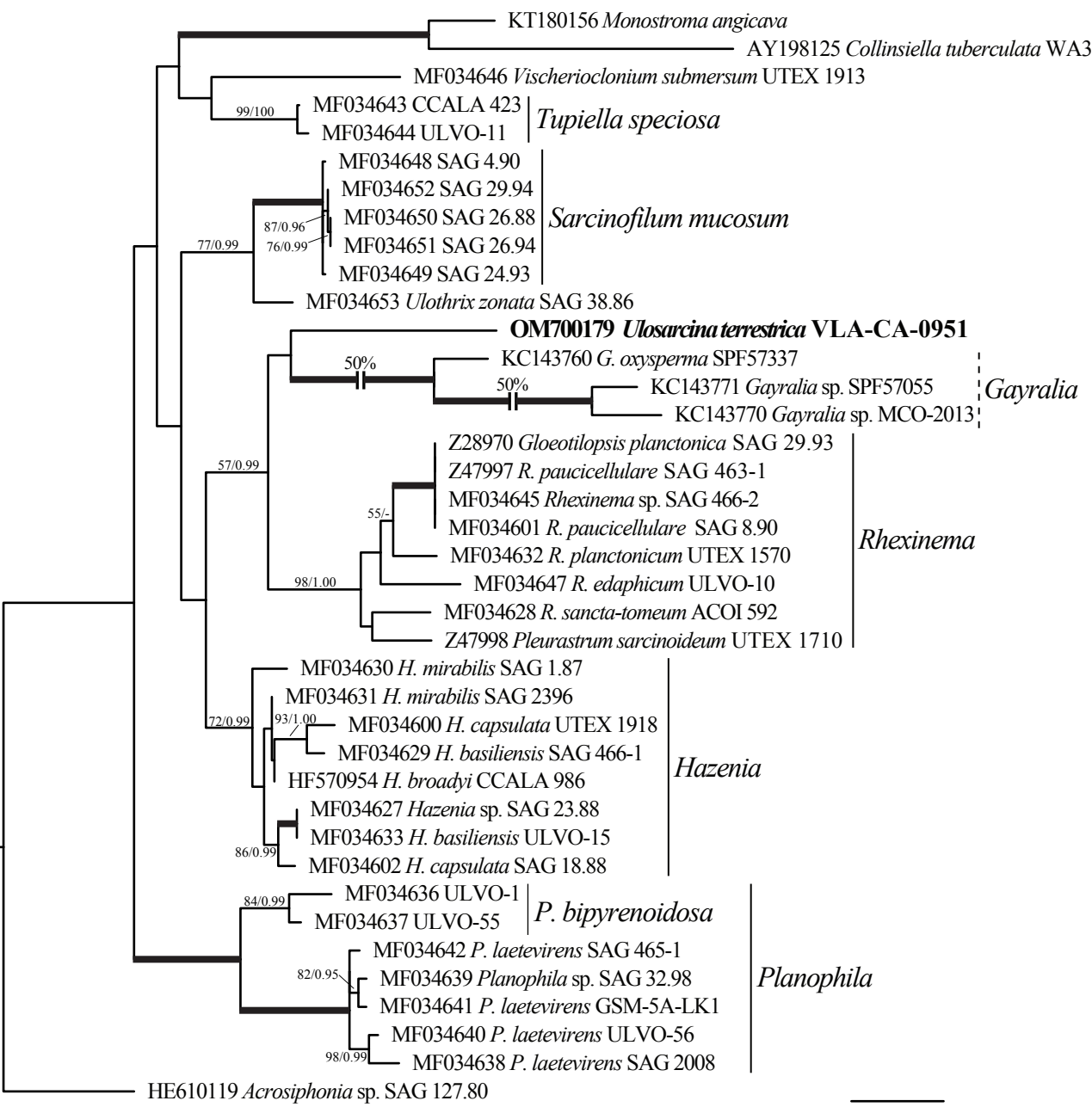

Supplement: Supplementary file 1 [file plants-11-03228-s001.zip › Fig_S2.pdf]

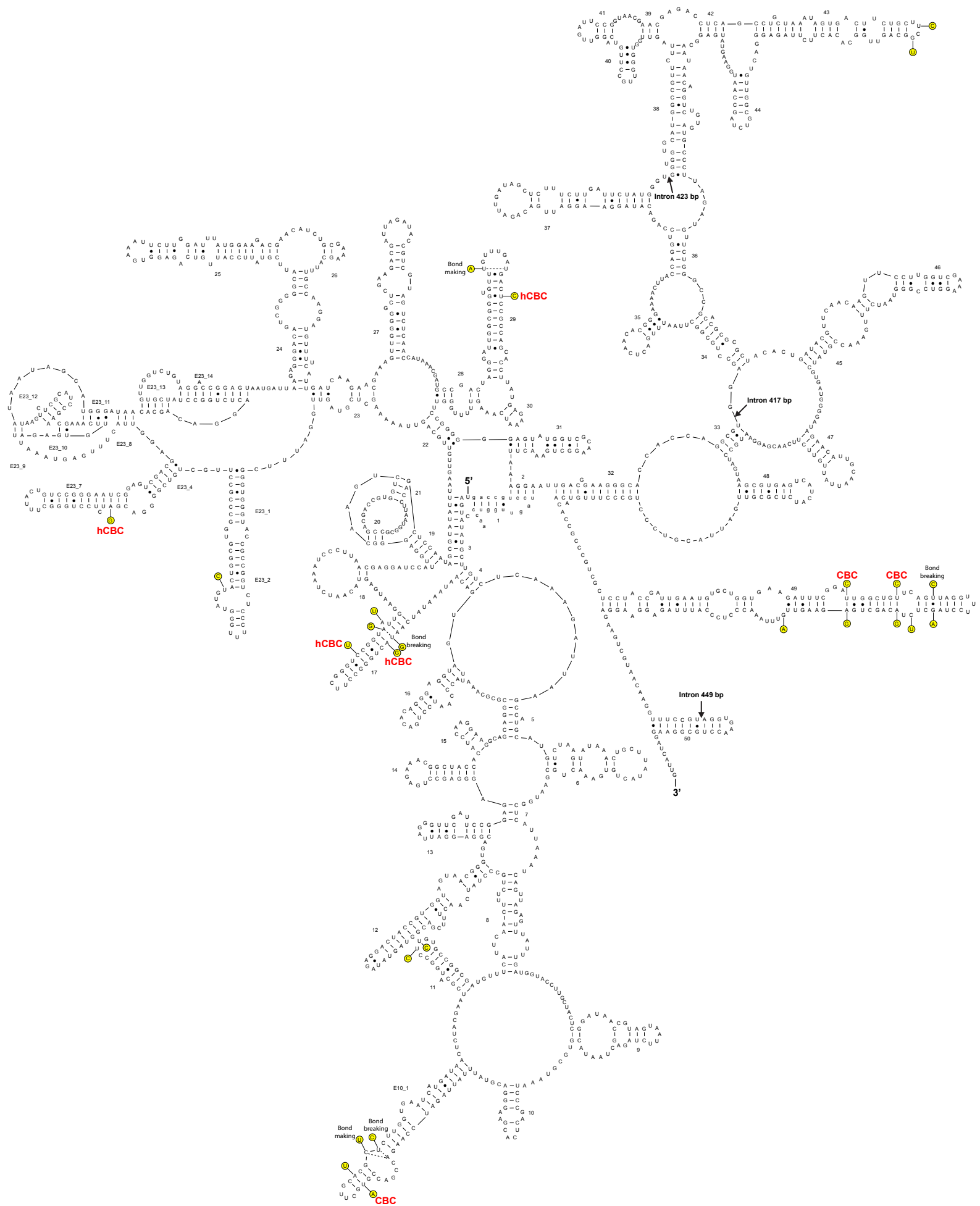

Supplement: Supplementary file 1 [file plants-11-03228-s001.zip › Fig_S3.pdf]

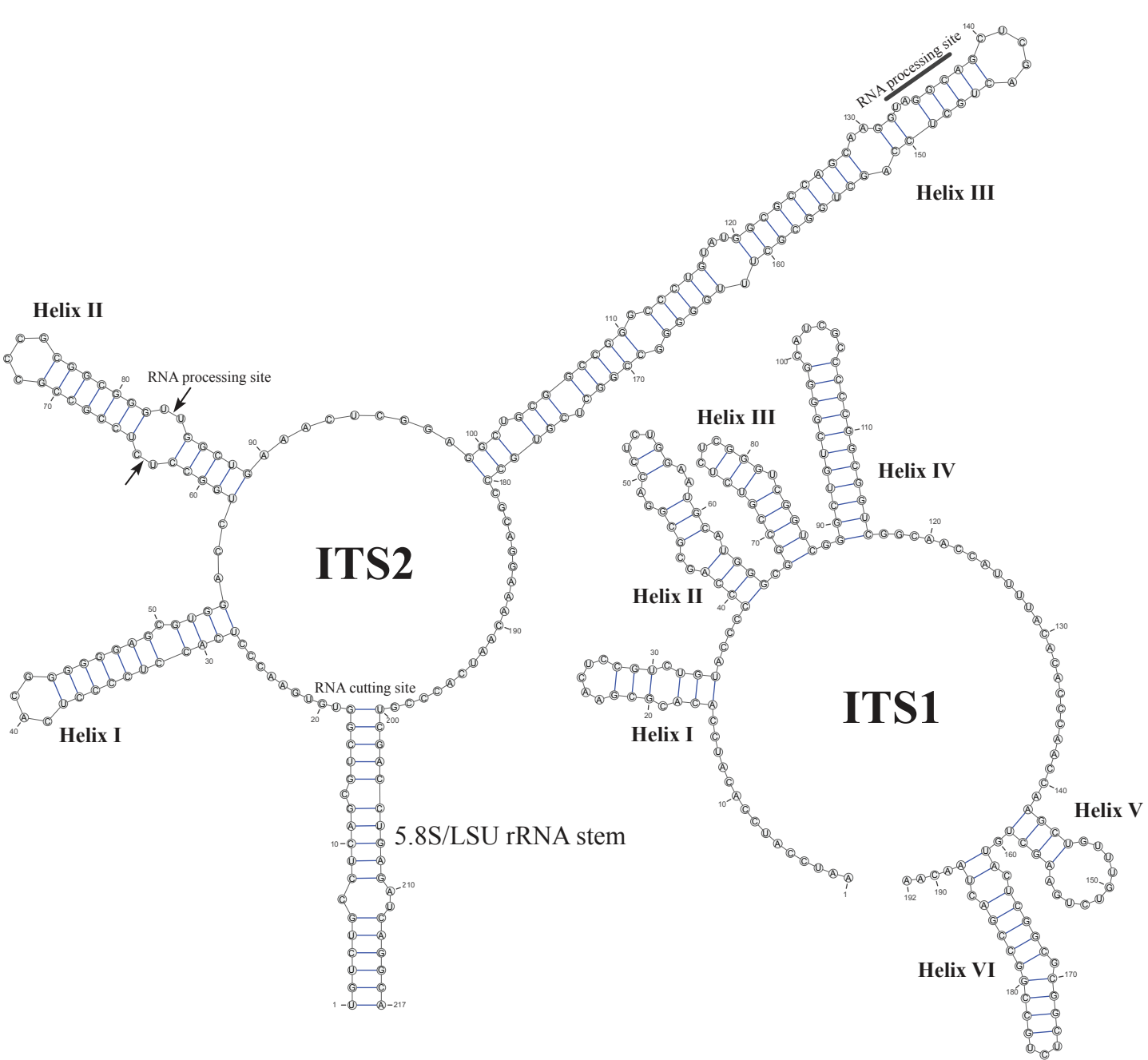

Supplement: Supplementary file 1 [file plants-11-03228-s001.zip › Fig_S4.pdf]
